# PRKDC-Mediated NHEJ May Play a Crucial Role in Aneuploidy of Chromosome 8-Driven Progression of Ovarian Cancer

**DOI:** 10.3390/ijms25094825

**Published:** 2024-04-28

**Authors:** Wenqing Luan, Hongyan Cheng, Haoling Xie, Huiping Liu, Yicheng Wang, Shang Wang, Xue Ye, Honglan Zhu, Fuchou Tang, Yi Li, Xiaohong Chang

**Affiliations:** 1 Department of Obstetrics and Gynecology, Peking University People’s Hospital, School of Life Sciences, Biomedical Pioneering Innovation Center, Peking University, Beijing 100044, China; 15650035912@163.com (W.L.); chenghongyanrenmin@163.com (H.C.); hlxie@pku.edu.cn (H.X.); yxylhp@126.com (H.L.); 1701110557@pku.edu.cn (Y.W.); 15822448522@163.com (S.W.); yexuemail@126.com (X.Y.); honglanzhu01@163.com (H.Z.); tangfuchou@pku.edu.cn (F.T.); 2 Center of Gynecologic Oncology, Peking University People’s Hospital, Beijing 100044, China; 3 Beijing Advanced Innovation Center for Genomics (ICG), Ministry of Education Key Laboratory of Cell Proliferation and Differentiation, Beijing 100871, China

**Keywords:** epithelial ovarian cancer, CNV, aneuploidy, NHEJ, PRKDC

## Abstract

High malignancy is a prominent characteristic of epithelial ovarian cancer (EOC), emphasizing the necessity for further elucidation of the potential mechanisms underlying cancer progression. Aneuploidy and copy number variation (CNV) partially contribute to the heightened malignancy observed in EOC; however, the precise features of aneuploidy and their underlying molecular patterns, as well as the relationship between CNV and aneuploidy in EOC, remain unclear. In this study, we employed single-cell sequencing data along with The Cancer Genome Atlas (TCGA) to investigate aneuploidy and CNV in EOC. The technique of fluorescence in situ hybridization (FISH) was employed using specific probes. The copy number variation within the genomic region of chromosome 8 (42754568-47889815) was assessed and utilized as a representative measure for the ploidy status of individual cells in chromosome 8. Differential expression analysis was performed between different subgroups based on chromosome 8 ploidy. Gene ontology (GO), Kyoto Encyclopedia of Genes and Genomes (KEGG), protein–protein interaction (PPI), and hub–gene analyses were subsequently utilized to identify crucial genes involved. By classifying enriched tumor cells into distinct subtypes based on chromosome 8 ploidy combined with TCGA data integration, we identified key genes driving chromosome 8 aneuploidy in EOC, revealing that *PRKDC* gene involvement through the mediated non-homologous end-joining pathway may play a pivotal role in disease progression. Further validation through analysis of the GEO and TCGA database and survival assessment, considering both mRNA expression levels and CNV status of *PRKDC*, has confirmed its involvement in the progression of EOC. Further functional analysis revealed an upregulation of *PRKDC* in both ovarian EOC cells and tissues, with its expression showing a significant correlation with the extent of copy number variation (CNV) on chromosome 8. Taken together, CNV amplification and aneuploidy of chromosome 8 are important characteristics of EOC. *PRKDC* and the mediated NHEJ pathway may play a crucial role in driving aneuploidy on chromosome 8 during the progression of EOC.

## 1. Introduction

Ovarian cancer (OC) is the leading cause of cancer mortality among gynecological malignancies worldwide. Ovarian cancer exhibits a complex histopathological pattern, with epithelial ovarian cancer comprising 85% to 90% of cases. Epithelial ovarian cancer encompasses at least five distinct malignant subtypes: high-grade serous carcinomas (HGSOC) account for approximately 70%, while low-grade serous carcinomas (LGSOC) represent around 5%. Endometrium of carcinoma (EnOC) constitutes about 10%, ovarian clear cell carcinoma (OCCC) ranges from 6% to 10%, and mucinous ovarian carcinoma (MOC) comprises roughly 3%, with the remaining being rare subtypes [1]. The high mortality rate of patients with ovarian cancer is primarily attributed to the highly malignant nature of this disease, characterized by aggressive metastasis, frequent disease recurrence, and resistance to multiple drugs. These factors collectively contribute to unfavorable patient outcomes [2]. The poor prognosis of EOC patients emphasizes the need for further insight into the potential mechanisms driving tumor malignancy. 

While previous studies have predominantly focused on gene mutations as pivotal molecular events in epithelial ovarian cancer, the significance of copy number variation (CNV) has garnered increasing attention. A previous investigation explored the genetic and epigenetic characteristics of cancer, leading to a hierarchical classification of 3299 TCGA tumor samples from 12 cancer types [3]. The findings revealed that distinct tumors are predominantly characterized by either mutations (M class) or copy number variations (C class). A majority of EOC were classified into class C, challenging the conventional understanding of EOC where mutations were considered more significant in tumorigenesis or tumor progression. Nevertheless, the underlying mechanisms responsible for inducing aneuploidy remain largely elusive. 

Copy number variations (CNVs) refer to duplications or deletions occurring in specific chromosomes, spanning regions ranging from 1 kb to several Mb, resulting in variable copy numbers compared to a reference genome [4]. While CNVs occur in both tumor and non-tumor cells at equal rates, the frequency and diversity of CNVs are higher in malignant tumors, with a greater prevalence of copy number amplifications compared to copy number deletions [5]. Genes mapping in proximity to copy number variations (CNVs) have been demonstrated to exhibit altered expression levels compared with transcripts lacking CNVs, thereby potentially leading to the upregulation of oncogenes [6]. Multiple studies have consistently demonstrated a robust association between copy number variations (CNVs) and unfavorable cancer prognosis [7,8,9]. The presence of multiple copy number variations (CNVs) in the genome can contribute to genomic heterogeneity, including aneuploidy and distinct molecular phenotypes. Tumor cells harboring CNVs are believed to arise from chromosome instability, including aneuploidy of chromosomes [10]. However, in the context of intra-tumor heterogeneity, the presence of high aneuploidy in a single cell does not necessarily imply a corresponding high level of CNV heterogeneity among tumor cells. Therefore, employing single-cell analysis may offer valuable insights into unraveling the underlying molecular mechanism driving aneuploidy and CNV alterations that promote tumor progression [11].

Over a century ago, Boveri identified aneuploidy as a hallmark of cancer. Aneuploidy, characterized by aberrations in the number and structure of chromosomes, represents a prevalent phenomenon observed in the majority of human malignancies. In certain malignancies, specific aneuploidies exhibit a higher frequency compared to mutations in well-established oncogenes or tumor suppressor genes [12]. Aneuploidy frequently emerges early in tumorigenesis, whereas several sequencing studies have unveiled that specific chromosomal alterations also manifest at later stages of tumor development [13,14]. Chromosome gains and losses typically result in a proportion alteration of gene expression on the affected chromosome, leading to the emergence of a specific aneuploidy phenotype that may confer selective advantages in certain environments [15]. Aneuploidy can result in the acquisition and loss of specific tumor suppressor genes and oncogenes, respectively, thereby leading to malignant transformation. Moreover, aneuploid cells possess the capacity to proliferate under sub-optimal conditions, thereby facilitating their dissemination to distant anatomical sites within the organism. Previous studies have demonstrated that cancer cells with trisomies exhibit a proliferative advantage when cultured under hypoxic or serum-free conditions, as compared to those harboring single additional chromosomes [16]. Additionally, aneuploidy confers cancer cells with the ability to adapt and survive in the presence of diverse anticancer agents and is strongly associated with resistance to specific drugs in human cancer cell lines exhibiting arm-length or numerical aneuploidies [17,18]. The presence of aneuploidy in tumors is also considered a prognostic indicator for poor outcomes, as it has been associated with aggressive characteristics such as metastatic dissemination and resistance to therapy. Therefore, aneuploidy has been proposed as a potential genetic driver of cancer development based on the “chromosome theory of cancer”. The relationship between copy number variations (CNV) and chromosome instability is intricate. Both CNV and chromosome instability can result in aberrant gene expression, and multiple lines of evidence highlight the pivotal role of imbalanced gene expression in understanding phenotypes induced by aneuploidy. Aneuploidy-induced alterations in copy numbers play a crucial role in driving aberrant gene expression. For instance, mouse models of Down syndrome are generally associated with a decrease in angiogenesis, and several genes affected by additional numbers of chromosome 21 are overexpressed, including *Dscr1*, *Col18a1*, and *Adamts1* genes, which typically reduce blood vessel formation. However, research on these aspects in epithelial ovarian cancer (EOC) is still in its nascent stages. The majority (approximately 90%) of human EOCs exhibit aneuploidy [19]. While the association between aneuploidy and malignancy has been well established, the most significant characteristic of aneuploidy in EOC, as well as the underlying molecular mechanisms driving aneuploidy and the relationship between copy number variations (CNV) and aneuploidy in EOC, remain elusive.

The fluorescence in situ hybridization (FISH) technique is a valuable approach for the analysis of chromosomal aberrations, specifically pertaining to changes in chromosome number. Numerous studies investigating aneuploidy in epithelial ovarian cancer (EOC) utilizing diverse FISH probes have unveiled multiple alterations within chromosomes [20,21]. The FISH analysis employing diverse probes targeting chromosomal centromeres has been extensively utilized for the detection of tumor aneuploidy. Chromosome 8 (Chr8) instability has long been a focal point in ovarian cancer research. The c-Myc gene, located at 8q24, is a prominent and versatile proto-oncogene with extensive research applications. It has been widely employed as a probe in numerous studies. Amplification of the Centromere probe for chromosome 8 (CEP8) and c-Myc has been observed in various human cancers, including EOC, and is associated with unfavorable prognosis. However, the detection of chromosome aneuploidy through FISH analysis is constrained by its limited throughput and imprecise results. Single-cell sequencing offers a robust tool for investigating aneuploidy, and our research team has previously developed a single-cell multi-omics sequencing technology known as scCOOL-seq2 [22]. In the system, CNVs, the DNA methylome, chromatin accessibility, and the transcriptome are simultaneously assessed in the same individual cell to reconstruct the genetic lineages of cancer cells. scCOOL-seq2 reveals both the CNVs from a more holistic perspective and further distinguishes aneuploid tumor cells, which may provide insights into understanding the underlying molecular mechanisms of aneuploidy-mediated tumor progression.

In this study, we investigated genome-wide copy number variations (CNVs) present in individual EOC cells. Among all the analyzed chromosomes, chromosome 8 (Chr8) exhibited the most significant overall amplification as well as within each individual cell. Fluorescence in situ hybridization (FISH) analysis confirmed that the numerical gain of Chr8 was a prominent feature observed in EOC samples. We also explored the clinical association between Chr8 gain or c-Myc/Chr8 ratio. To further elucidate the underlying mechanisms driving aneuploidy of chromosome 8 and CNVs during the promotion process of epithelial ovarian cancer progression, we enriched single tumor cells and sub-classified them into diploid and polyploid cells based on DNA content within segments from regions 8p11.1-8q11.l on chromosome 8. We identified differentially expressed genes (DEGs) between Chr8 polyploid cells and non-tumor cells and determined that the *PRKDC* gene plays a crucial role in promoting the gain of Chr8 in EOC. The gain of Chr8 in EOC was further confirmed through the analysis of TCGA database. Additionally, among all copy-number amplified genes on chromosome 8, *PRKDC* was identified as a hub gene. Further investigation into *PRKDC* revealed its significant role in driving aneuploidy of Chr8 and promoting the progression of EOC.

## 2. Results

### 2.1. Multiple CNVs on Chr8 in EOC Were Found through Single-Cell Multi-Omics Sequencing and TCGA Database

**Figure 1:**
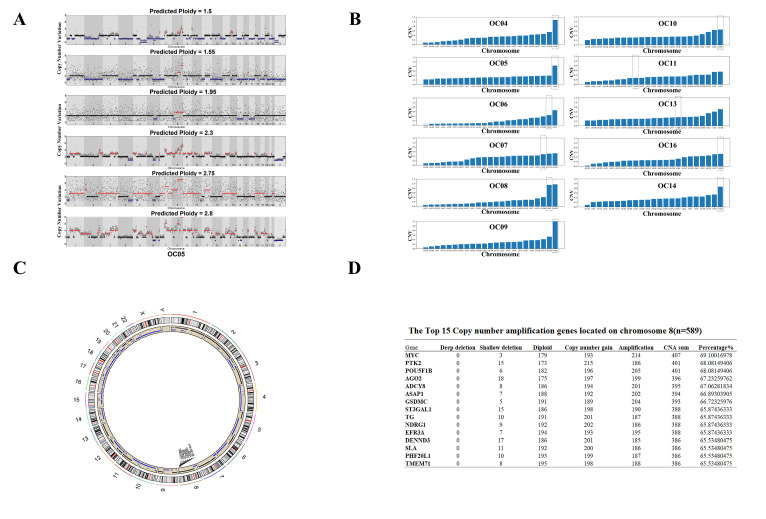
Multiple CNV amplification in Chromosome 8 was found through single-cell multi-omics sequencing and TCGA database. (**A**) Genomic sequencing analysis of each single enriched tumor cell by single-cell sequencing. Each row represents a single cell. The color scale ranges from blue (deletion) to red (amplification). (**B**) Copy Number Variation analysis of different chromosomes in each patient by single-cell sequencing. The y-coordinate is the frequency of CNV, and the horizontal coordinate represents the different chromosomes. (**C**) The Circos of the human genome showing chromosome structure and location of CNV (amplification and deletion) in ovarian cancer. The outermost layer was the chromosome model, and the next demonstrated the CNV. Different colors, as noted in the center of the Circos, represent different genomic alterations. (**D**) The top 15 copy number amplification genes located on chromosome 8 (n = 589). The CNV status was defined by GISTIC, including Deep deletion (homozygous deletion), Shallow deletion (heterozygous deletion), Diploid (normal copy number), Copy number gain (low-level gain), and Amplification (high-level amplification).

The detailed methods of single-cell sequencing are described in our previous studies [22]. Cells that met the criteria as defined in these methods were screened; nuclear fractions of these cells were sequenced, and DNA profiling was performed to select the cells with clear SCNA patterns deduced from the genomic sequencing data. The results revealed amplification on some chromosomes in EOC cells. Furthermore, we found that the most pronounced amplification occurred on Chr8. Next, we used scCOOL-seq2 DNA data to reconstruct the CNVs on each chromosome of each enriched single tumor cell and confirmed this observation (Figure 1A). We examined the CNVs in each individual patient and found that the highest degree and the highest number of CNVs was on Chr8 (Figure 1B). A Circos plot was used to display the genomic alterations in EOC, including CNVs (Figure 1C). The occurrence of copy number variations (CNVs) was frequently observed in epithelial ovarian cancer (EOC), exhibiting an uneven distribution across almost all chromosomes. Notably, marked amplification of copy numbers was detected on Chromosome 8. The genes exhibiting the highest frequency of copy number variations (CNVs) in the 589 samples of epithelial ovarian cancer (EOC) are depicted in Figure 1D. Notably, the *MYC* gene, a well-established oncogene, demonstrates the most frequent CNV events (407/589, 69.10%), followed by the *PTK2* gene (401/589, 68.08%). These findings strongly suggest that *MYC* plays a crucial role in driving chromosome instability in EOC. Collectively, these results underscore the significance of Chr8 aneuploidy in EOC.

### 2.2. Aneuploidy of Chr8 Is Present in EOC Tissues, Cell Lines and CTCs

Single-cell sequencing and TCGA database analysis both showed that copy number amplification significantly occurred in most regions of Chr8. To further confirm whether Chr8 aneuploidy was present in EOC, we performed FISH analysis with a probe for CEP8, which detects regions on Chr8, in five EOC cell lines (A2780, SKOV-3, CAOV3, ES2, and SKOV3-IP) and an ovarian benign neoplasm endometriosis cell line (hEM15A) as a control.

**Figure 2:**
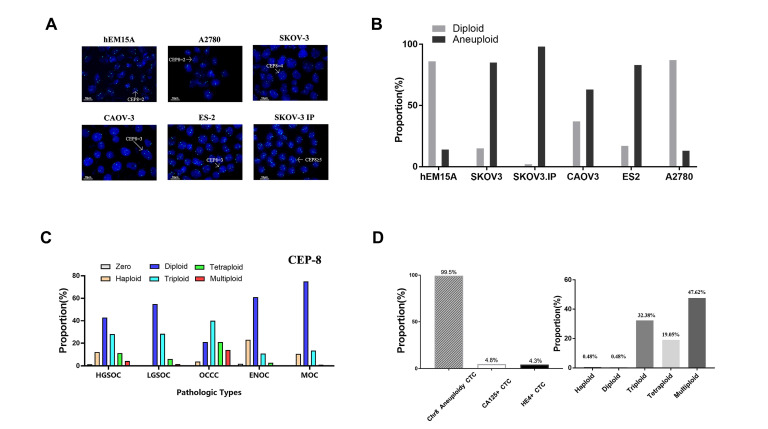
Aneuploidy of Chromosome 8 was identified as an important character by Fluorescence in situ hybridization (FISH) with probes in ovarian cancer tissue cell lines and CTCs. (**A**) Representative patterns of FISH of chromosome 8 (CEP8) (blue color) copy number status. (**B**) The proportion of different ploidy of chromosomes 8 detected by CEP8 FISH in different ovarian cancer cell lines. Endometriosis cell line hem15A was used as a control. (**C**) The proportion of different ploidy of chromosomes 8 detected by CEP8 FISH in different pathological types of ovarian cancer. (**D**) The left represents the distribution of CA125 and HE4 expression and Chr8 aneuploidy in CTCs detected by SE-iFISH in 20 ovarian cancer patients. The right represents the proportion of different ploidy of chromosomes 8.

Figure 2A shows the representative patterns of FISH staining for Chr8. As shown in Figure 2B, almost all hEM15A (control) and A2780 (EOC) cells were Chr8 diploid cells; in the other EOC cell lines, Chr8 aneuploidy cells were dominant. The proportion of different ploidy status of Chr8 in every cell line is shown in Figure S1F. 

The results showed that the Chr8 aneuploidy status in EOC cell lines is mainly triploid and tetraploid. Chr8 tetraploid cells predominated in SKOV3 and SKOV3-IP cell lines, while Chr8 triploid cells were the largest proportion of cells in ES2 and CAOV3. 

To examine the clinical significance of Chr8 aneuploidy and copy number amplified genes in EOC, FISH analysis of clinical tissue samples was conducted. The characteristics of the 32 patients in this cohort are summarized in Table 1. 

In addition to the CEP8 probe, a probe for MYC was also used as a potential marker and a clinical diagnostic tool, as used in our previous research. Figure 2A and Figure S1D represent images of CEP8-FISH for detecting cell lines and c-Myc-FISH for detecting tumor tissues. We counted 100 cells per sample from each patient. Figure 2C and Figure S1G show the proportion of different copy numbers of Chr8 and c-Myc in different pathological patterns. A corresponding algorithm was used to evaluate the average copy number of tumor samples per patient. The copy number of CEP8 ranged from 1.53 to 4.08 per patient, while c-Myc ranged from 1.47 up to 6.99. The results indicated that copy number amplification of Chr8 was present in almost all pathological types of EOC. Because the *c-Myc* gene was located on Chr8, Spearman correlation analysis was used to investigate the correlation of the copy number of CEP8 and c-Myc in a single nucleus. As shown in Figure S1H, there was a slight positive correlation between CEP8-FISH and c-Myc-FISH (r = 0.36, *p* < 0.001). Figure S1I shows the ratio of c-Myc/CEP8 in different pathological types of EOC, and the results are the same as those of CEP8 (Figure 2C).

The median copy number of CEP8 in tumor tissue was 2.36, and this was used as a cut-off value to distinguish between a high degree and a low degree of Chr8 aneuploidy, as shown in Table 2.

Chr8 aneuploidy was not associated with patient age, pathological pattern, stage, grade, lymphatic metastasis, ascites, or status of ER or p53. We defined c-Myc amplification as ≥1.5 copies of c-Myc/CEP8 per cell and found that c-Myc amplification was associated with the pathological pattern, grade, lymphatic metastasis, and ER positivity (Table 3). 

Circulating tumor cells (CTCs) in peripheral blood were enriched by SE-iFISH, as described in our previous study [23]. A total of 210 CTCs were enriched from 20 EOC patients. Positive expression of one of three CTC markers (CEP8, CA125, HE4) was considered to indicate CTCs [24]. Figure S1E shows representative images of chromosomal aneuploidy in CTCs detected by the SE-iFISH platform. Almost all 120 CTCs showed aneuploidy of Chr8. Subclassifying CTCs on the basis of cell size revealed that cell size was positively correlated with Chr8 ploidy number (Figure 2D).

### 2.3. Screening Key Genes Driving Chr8 Aneuploidy in EOC through Classifying Enriched Single Tumor Cells by Single-Cell Sequencing Method into Different Ploidy of Chr8 Subtypes

**Figure 3:**
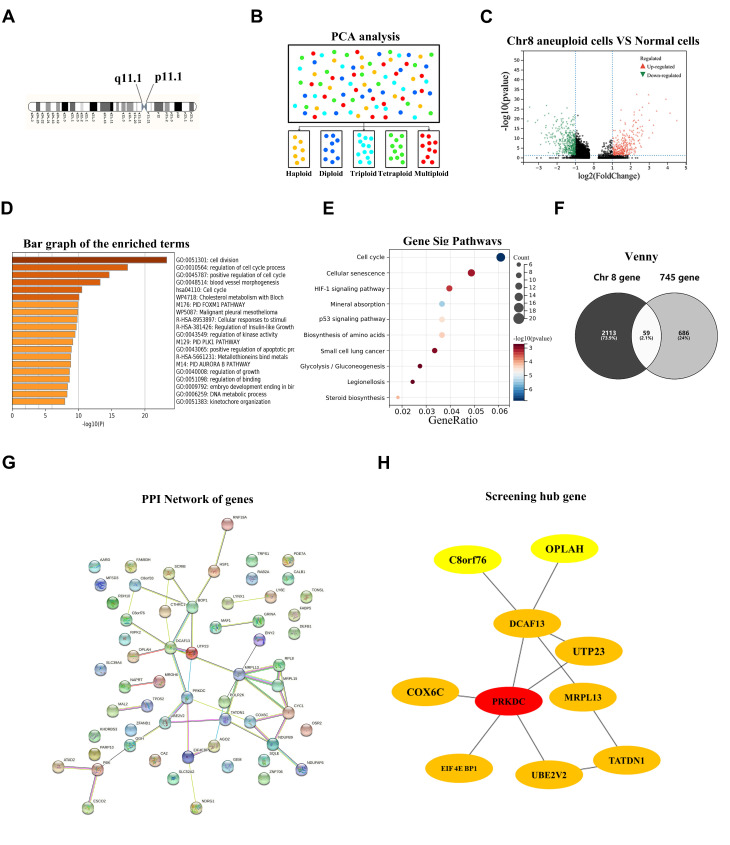
Screening key genes driving chromosome 8 aneuploidy in ovarian cancer through classifying enriched single tumor cells in the single-cell sequencing method into different ploidy of chromosome 8 subtypes. (**A**) Schematic diagram of Classification criteria for single-cell sequencing enriched cells. Chromosome 8p11.1-8q11.1 section was selected as a standard for distinguishing enriched single cells with different ploidy of chromosome 8. (**B**) Results of principal component analysis (PCA) of all cancer cells we defined show the different ploidy of chromosome 8 subtypes. Different colors represent different patients, which is the same as in Figure 1A. (**C**) The volcano plot of mRNA levels from the single-cell sequencing and the x-axis represent the log2 transformed of fold change ratios. The y-axis is the log10 transformed adjusted p-value. The red colored dots represent the DEGs based on fold change > 1. Herein, the volcano plot displayed the different genes when comparing Chr8 polyploidy cancer cells with the normal cells. (**D**) Venn diagram representing the distribution of DEGs in different groups; 59 DEGs were upregulated in both scenarios of genes located on Chr8 and Chr8 polyploidy tumor cells vs. normal cells. (**E**) GO analysis of 745 upregulated genes in Chr8 polyploidy tumor cells group. (**F**) KEGG analysis of 745 upregulated genes in Chr8 polyploidy tumor cells group. (**G**) A protein–protein interaction (PPI) network shows the interaction between the screened DEGs. Each node represents one gene; the edge indicates the interaction relationship. (**H**) The top 10 genes in the PPI network were identified as central genes using EcCentricity.

Single-cell sequencing not only enables the discrimination of tumor cells from non-tumor cells but also facilitates comprehensive omics analysis on individual tumor cells, thereby facilitating the characterization of redefined cell subtypes. To ensure consistency with FISH analysis conducted on cancer cell lines and tissues, we specifically targeted the Chr8p11.1–8q11.1 region bounded by the Chr8 centromere probe (Figure 3A). A total of 10 patients had both CEP8-FISH analysis and single-cell sequencing data available for comparison. The proportion of cells exhibiting different ploidy levels in chromosome 8 was assessed based on various criteria. As depicted in Figure S1A–C, these results further validated the accuracy of subclass analysis performed using single-cell sequencing data. Principal component analysis (PCA) was employed to categorize cancer cells into distinct groups based on their Chr8 ploidy status. As depicted in Figure 3B, cells exhibiting a ploidy of ≥5 were identified as multiploidy cells, and differentially expressed genes (DEGs) were further analyzed between Chr8 polyploidy tumor cells (including Chr8 triploid, tetraploid, and multiploidy tumor cells) and non-tumor cells (Figure 3C). A total of 745 upregulated genes (defined as fold change > 1) were identified. Subsequently, Gene Ontology (GO) analysis and Kyoto Encyclopedia of Genes and Genomes (KEGG) pathway analyses revealed that the set of 745 upregulated genes was predominantly enriched in GO terms associated with cell cycle regulation and division processes (Figure 3D). Moreover, these genes exhibited significant associations with cellular pathways such as the cell cycle signaling pathway and cell senescence pathway (Figure 3E). In order to investigate potential drivers for Chr8 aneuploidy in EOC, we initially screened for genes located on Chr8 among the pool of 745 upregulated genes. As illustrated in Figure 3F, a total of 59 upregulated genes within Chr8 polyploidy tumor cells were found to be situated on Chr8. Furthermore, we constructed a protein interaction network using these aforementioned proteins through the STRING database analysis method (Figure 3G). By employing Cytoscape software to identify hub genes based on EcCentricity calculations, we discovered ten hub genes, including *PRKDC, UBE2V2, DCAF13, EIF4EBP1*, and *TATDN1* genes (Figure 3H). 

### 2.4. Screening Key Genes Driving Chr8 Aneuploidy in EOC through TCGA Database with CNV, Clinical Data, and mRNA of 589 Patients

**Figure 4:**
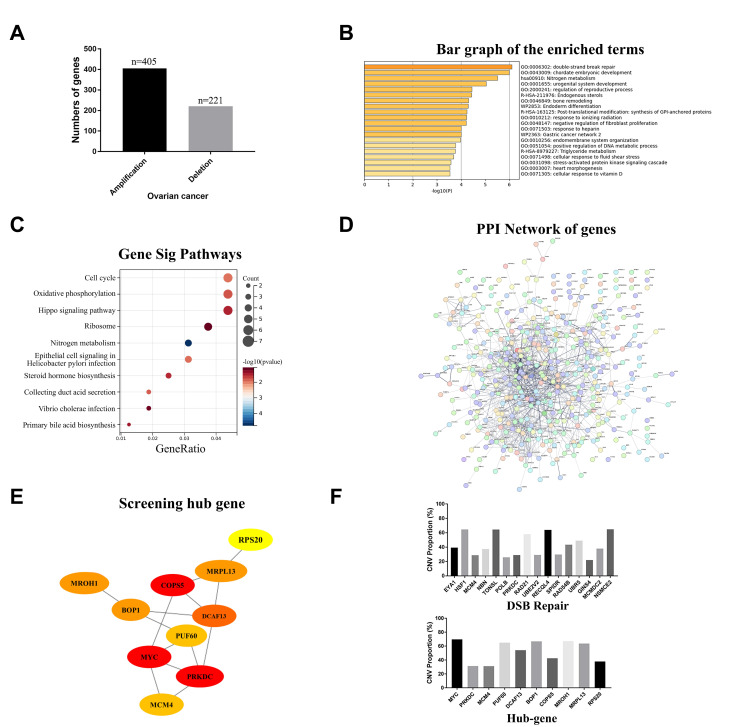
Screening key genes driving chromosome 8 aneuploidy in ovarian cancer through TCGA database with CNV, clinical data, and mRNA of 589 patients. (**A**) Events of copy number loss or gain of genes located on chromosome 8 in ovarian cancer samples. (**B**) Bar graph of the enriched terms (the top 20 clusters) and the top-ranked Gene Ontology biological processes. (**C**) GO analysis of 405 amplified gene located on chr8. (**D**) A protein–protein interaction (PPI) network shows the interaction between 405 amplified genes located on chr8. (**E**) The top 10 genes in the PPI network were identified as central genes using Closeness. (**F**) Percentage of EOC samples with CNVs of hub genes and double-strand break repair genes through Closeness according to TCGA database.

The CNVs located on Chr8 were frequently observed in the 589 EOC samples in TCGA database. As depicted in Figure 4A, among the 2172 genes situated on Chr8 (NCBI Homo sapiens Annotation Release 109.20210226), CNVs were detected in 626 genes across the 589 samples, resulting in a total of 195740 CNV events identified on Chr8. For each gene, copy number “gain” was defined when the counts of copy number gain (+1) and amplification (+2) exceeded those of deep deletion (−2) and shallow deletion (−1) within the 589 samples. Ultimately, a set of 405 genes was designated as exhibiting “gain”. Subsequently, Metascape analysis was conducted to investigate these selected genes, revealing significant enrichment in double-strand break repair and chordate embryonic development processes (Figure 4B). Moreover, the Kyoto Encyclopedia of Genes and Genomes (KEGG) enrichment analysis demonstrated that these same genes exhibited notable enrichment within the cell cycle pathway (Figure 4C). Additionally, a protein–protein interaction enrichment analysis was performed to further explore their functional associations (Figure 4D). Hub genes were identified by Cytoscape (Figure 4E). Notably, *PRKDC* ranked first among all 10 hub genes, similar to its identification as an enriched hub gene generated from single-cell sequencing data. Figure 4F shows the proportion of CNV during the hub gene list and double-strand breaking repair gene terms. We further evaluated the relationship between the alterations (CNVs) of the *PRKDC* gene and the clinicopathological features of EOC patients. The analysis included a total of 578 samples after excluding those with insufficient clinical information. The results showed that CNV of the *PRKDC* gene was associated with age, pathological stage, FIGO stage, and venous and lymphatic invasion (Table 4). 

### 2.5. The PRKDC Gene May Play a Crucial Role in Chr8 Aneuploidy in EOC to Promote Tumor Progression

**Figure 5:**
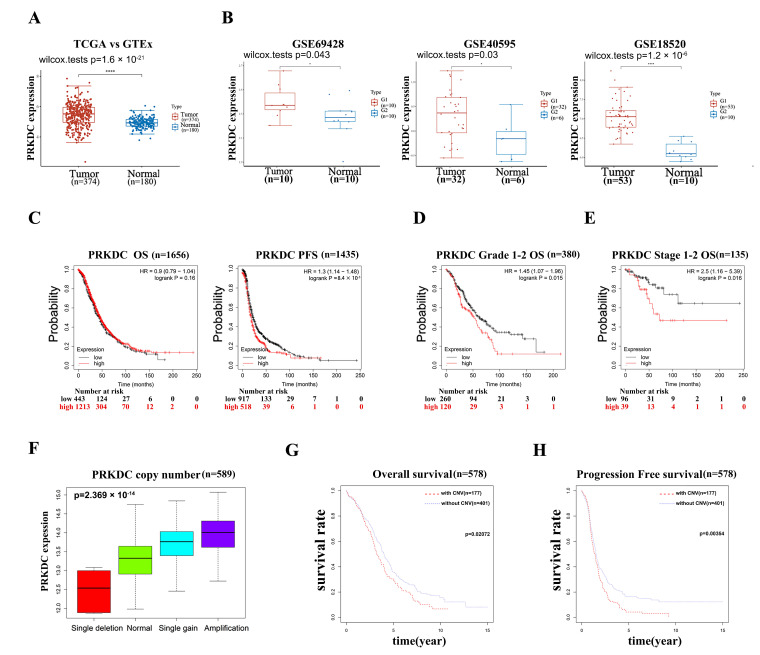
PRKDC CNA may play a crucial role in chromosome 8 aneuploidy in ovarian cancer to promote the progress of the tumor. (**A**) mRNA expression of *PRKDC* in cancer vs. control from patients in TCGA database. (**B**) Validation of *PRKDC* expression from 3 GEO databases, GSE69428, GSE40595, and GSE18520. The mRNA level of *PRKDC* was analyzed with the “limma” package using the Student’s t-test. (**C**) Kaplan–Meier survival curves of the mRNA expression level of *PRKDC* for all ovarian cancer patients (n = 1656). (**D**) Kaplan–Meier survival curves of the mRNA expression level of *PRKDC* for low-grade (grade 1+2) ovarian cancer patients (n = 380). (**E**) Kaplan–Meier survival curves of the mRNA expression level of *PRKDC* for early-stage (FIGOI+II) ovarian cancer patients (n = 135). (**F**) mRNA expression levels of *PRKDC* in different CNV patterns. (**G**) Kaplan–Meier curves for overall survival of EOC patients (n = 578) in TCGA according to the presence or absence of CNV of *PRKDC*. (**H**) Kaplan–Meier curves for progression-free survival of EOC patients (n = 578) in TCGA according to the presence or absence of CNV of *PRKDC*. * means *p* < 0.05 and **** means *p* < 0.0001.

From the results showing that *PRKDC* was in the list of hub genes generated from both our single-cell sequencing data after subclassifying cancer cells by Chr8p11.1–8q11.1 and TCGA database with CNV data and corresponding clinical data, we speculated whether *PRKDC* expression was altered in EOC development through Chr8 aneuploidy. We first evaluated *PRKDC* expression in EOC. We compared *PRKDC* mRNA expression in 374 EOC samples from TCGA database and 180 normal ovarian tissues from the GTEx database as the control. As shown in Figure 5A, *PRKDC* mRNA expression was significantly upregulated in EOC compared with that in control tissues. *PRKDC* mRNA expression in EOC was higher than that in normal FTE or OSE tissues (Figure 5B). The prognostic value of *PRKDC* in patients with EOC was further investigated using the Kaplan–Meier plotter website. The expression level of *PRKDC* was not associated with overall survival (OS) (HR, 0.9; 95% CI, 0.79–1.04; *p* = 0.16), whereas it was associated with progression-free survival (PFS) (HR, 1.3; 95% CI, 1.14–1.48; *p* < 0.001) (Figure 5C). The prognostic values of *PRKDC* within the different pathology grades or FIGO stages of EOC were determined. As shown in Figure 5D,E, high expression of *PRKDC* mRNA was significantly associated with shorter OS in patients with FIGO stage I–II (HR, 1.45; 95% CI, 1.07–1.96; *p* = 0.015) or in patients with lower grades (HR, 2.5; 95% CI, 1.16–5.39; *p* = 0.016). 

Next, we evaluated the effect of alterations in *PRKDC* genes on *PRKDC* mRNA expression level. A total of 589 samples from TCGA database were included in the analysis. As expected, the mRNA levels were significantly associated with different CNV patterns. Deep or shallow deletions resulted in lower mRNA expression, while amplifications or copy number gains were related to higher mRNA expression (Figure 5F). We performed Kaplan–Meier analysis to investigate the impact of genetic alterations in *PRKDC* on overall survival (OS) and progression-free survival (PFS); the analysis included a total of 578 samples from TCGA database after excluding those with insufficient clinical information, as shown in Figure 5G,H, and found that patients with CNV of *PRKDC* gene had worse OS (*p* = 0.021) and DFS (*p* = 0.00354). 

### 2.6. PRKDC Was Upregulated in EOC Cells and Tissues, and the Expression of PRKDC Was Associated with Chromosome 8 CNV

**Figure 6:**
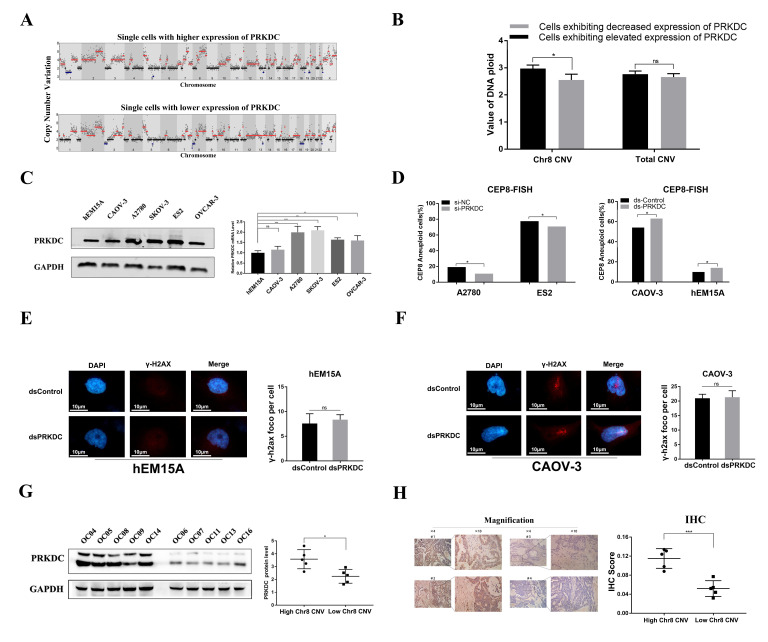
*PRKDC* was upregulated in ovarian cancer cells and tissues, and its expression was correlated with chromosome 8 CNV. (**A**) Representative images exhibit the copy number variation of each single cell of the group with elevated and decreased expression of *PRKDC*. (**B**) The significance of DNA ploidy in chromosome 8 copy number variation (CNV) or overall CNV assessment. (**C**) qRT-PCR and western blot assays were used to detect the expression of *PRKDC* in 5 ovarian cancer cell lines and ovarian benign tumor cell line hEM15A. (**D**) The proportion of CEP8 aneuploid cells after transfected with si*PRKDC* or ds*PRKDC* in EOC cell lines. (**E**) Representative confocal immunofluorescent images of γ–H2AX foci in hEM15A after being transfected with saRNA. DNA damage is determined by the ratio of foci count per cell (FPC) after being transfected with saRNA. (**F**) Representative confocal immunofluorescent images of γ–H2AX foci in CAOV-3 after being transfected with saRNA. DNA damage determined by the ratio of foci count per cell (FPC) after being transfected with saRNA. (**G**) Western blot assay was used to detect the expression of *PRKDC* in 10 tissue samples of each enrolled patient (left), and quantitation of western blot assay bands was performed using Image J 1.5.4 (right). (**H**) Immunohistochemical staining was used to detect the expression of *PRKDC* in 10 tissue samples of each enrolled patient (left), and the intensities of IHC staining were quantitated by Image-Pro Plus 6.0 (right). * means *p* < 0.05; ** means *p* < 0.01; *** means *p* < 0.001.

In order to distinguish whether *PRKDC* influenced chr8 CNV in ovarian cancer, enriched single cancer cells from single-cell sequencing were divided into two groups according to the expression of *PRKDC*. The copy number variation on each chromosome and the total genome of each group were subsequently subjected to analysis. Representative images of groups exhibiting elevated and decreased *PRKDC* expression are exhibited (Figure 6A). The results revealed a positive correlation between the expression level of *PRKDC* and the extent of copy number variations on chr8 in cancer cells (2.95 vs. 2.60, *p* = 0.031) (Figure 6B). In order to investigate the broader spectrum of this instability, we conducted a comprehensive analysis of the overall chromosomal alterations in two groups. The results showed that there was no statistically significant difference in total DNA ploidy values between the two groups, which indicated that the expression of *PRKDC* did not exert a significant impact on the copy number alterations across the entire genome. 

Subsequently, CEP8-FISH analysis was performed in siRPKDC cells and negative control cells, as well as dsPRKDC cells and negative dsControl cells. Initially, the expression of PRKDC was assessed in five epithelial ovarian cancer (EOC) cell lines. The results showed that the mRNA and protein of *PRKDC* were upregulated in EOC cell lines compared with hEM15A, a kind of benign ovarian tumor cell lineage (Figure 6C). The expression of *PRKDC* was relatively high in A2780 and ES2 cells, whereas CAOV-3 and hEM15A exhibited comparatively low levels of *PRKDC* expression. Therefore, A2780 and ES2 cell lines were selected for knockdown experiments, while hEM15A and CAOV-3 cell lines were chosen for overexpression experiments. The mRNA and protein levels of *PRKDC* in different cells were detected after transfected with siRNA and saRNA. The results indicated that compared with siNC and dsControl groups, siPRKDC and dsPRKDC, respectively, cause a significant reduction and induction in *PRKDC* mRNA (Figure S2A,D). This reduction and induction were further verified by Immunoblot (Figure S2B,C,E,F). Subsequently, results indicated that *PRKDC* siRNA was found to decrease the proportion of CEP8 aneuploid cells in A2780 and ES2 cells, while overexpression of *PRKDC* was found to increase the proportion of CEP8 aneuploid cells in CAOV-3 and hEM15A cells (Figure 6D). Both functional experiments indicated that an imbalance of *PRKDC* was related to chr8 CNV in epithelial ovarian cancer. Similarly, in order to ascertain that the impact of *PRKDC* on chromosome 8 copy number alteration was not attributed to its influence on the entire genome, we assessed DNA damage in cells subsequent to modulation of *PRKDC* expression as a reflection of global genomic instability. *PRKDC*-induced γ–H2AX foci formation, which suggests accumulation of DNA damage, was investigated. The results demonstrated no statistically significant disparity in the quantities of γ–H2AX foci per cell between the two experimental groups (Figure 6E,F). 

To investigate the impact of chr8 CNV on *PRKDC* expression, we categorized 10 patients from our single-cell sequencing into two groups based on their degree of CNV on chromosome 8 (Figure 1B). The expression of *PRKDC* was detected in these two groups. Figure 6G showed that the *PRKDC* protein level was significantly upregulated in a high degree of the chr8 CNV group (OC04, OC05, OC08, OC09, OC14) compared with a low degree of the chr8 CNV group (OC06, OC07, OC11, OC13, OC16). Consistent with our western-blot result, the expression of *PRKDC* detected by immunohistochemistry (IHC) was also upregulated in a high degree of the chr8 CNV group (Figure 6H). The data revealed a positive correlation between the degree of chr8 CNV and the expression level of *PRKDC*, providing further evidence that amplification of chromosome 8 copy number contributes to the upregulation of *PRKDC* expression.

## 3. Discussion

In this study, we observed a high prevalence of copy number amplification and aneuploidy on Chromosome 8 (Chr8) in epithelial ovarian cancer (EOC), as determined by scCOOL-seq2 and FISH techniques. Additionally, we performed further analysis using CNV data from TCGA database and generated a Circos plot to visually depict the chromosomal structure and location of CNVs (amplifications or deletions). This comprehensive approach provided additional evidence supporting the characteristic copy number amplification on Chr8 in EOC.

As we previously mentioned [3], the focus of this study lies in the characterization of genomic alterations in EOC. Although it is generally accepted that copy number variations (CNVs) are a subtype of structural genome variants, the definition of CNV is dynamically evolving, and the mechanism of CNV formation remains under investigation. Recent studies have demonstrated that the term CNV should not be limited to variants of “considerable length”, while it is essential to apply core criteria encompassing molecular mechanisms and the number and length of repeated elements to all variants [25]. Thus, the definition of CNVs can be extended to include several other variant types. On one end of the spectrum, there are changes involving entire sets of chromosomes and aneuploidies (i.e., numerical abnormalities defined as a loss or gain of an entire chromosome), as well as structural abnormalities represented as non-balanced chromosome rearrangements. Furthermore, several studies have demonstrated that chromosomal abnormalities can lead to copy number variations (CNVs) [26].

While several studies have demonstrated that aneuploidy can impair cellular fitness and increase susceptibility to survival stress [16], it has also been established that chromosomal instability confers advantages for tumor evolution. Cancer manifests aneuploid phenotypes, which are determined by the impact of altered gene copy numbers on the transcriptome and proteome [27]. Changes in gene expression caused by aneuploidy can result in certain phenotypes that are conducive to or promote cancer progression. Large-scale genomic analysis of tumor samples has begun to reveal the hidden order behind the aneuploidy-associated carcinogenetic process during cancer evolution. During the early stages, chromosome instability can result in a rapid increase of diverse aneuploid cell populations. As the environment changes, subsets of cells with the most advantageous karyotypes become predominant. For instance, the loss of the p arm of chromosome 9 is a highly selected driving event in clear-cell renal cell carcinoma metastasis [28]. In breast cancer, aneuploid subpopulations were found to expand from the primary breast tumor and subsequently seed liver metastasis [29]. Aneuploidy has also been associated with cell state transitions in EOC: loss of chromosome arm 16p correlates with an epithelial–mesenchymal transition that facilitates cancer cell dissemination, while the loss of chromosome arm 10p correlates with a mesenchymal–epithelial transition that promotes cellular outgrowth [30].

In epithelial ovarian cancer, additional copy numbers of Chr8 or genomic regions of focal and recurrent copy number alterations in Chr8q have been identified. The clinical relevance of Chr8 has been researched. Comparative genomic hybridization (CGH) with a resolution of approximately 0.8 Mb was applied in 28 primary ovarian tumors, and the results showed that gain of Chr8q13.2 occurred at a high frequency in EOC, especially in serous and late-stage tumors, suggesting that alternative mechanisms of genomic instability may play a role in this tumor type [31]. In a previous study of unusual responders, Lheureux identified Chr8 amplification as a common genomic feature in long responders to weekly paclitaxel among EOC patients, which suggests that Chr8 amplification may play a crucial role in multi-drug resistance [32]. The prognostic value of Chr8 amplification was further confirmed by FISH analysis, as previously mentioned. In this study, we utilized single-cell multi-omics sequencing technology (scCOOL-seq2) to identify copy number variations (CNVs) and aneuploidy of Chr8, which emerged as crucial characteristics of EOC. Our approach provided high-throughput and accurate results.

While differences in the growth rate, metabolism, cell cycle kinetics, and cell size have been observed in aneuploid cells in vitro, the impact of a single extra specific chromosome on cellular function and even the evolution of cancer is still unclear. The consequences of aneuploidy, like its causes, are often multifaceted and likely to be context-dependent. Whole-genome cDNA libraries, CRISPR technology, and RNAi libraries have made it possible to eliminate or modify any specific gene within the genome to uncover their effects on cancer-related phenotypes. In contrast, manipulating individual chromosomes or segments via chromosome-engineering strategies is considerably more challenging. For instance, while certain genes may be classified as oncogenes, precise definitions for cancer-specific abnormalities of chromosomes are yet to be established. Recent research has utilized microcell-mediated chromosome transfer approaches [33], and CRISPR-mediated chromosome elimination techniques have yielded some interesting results. Gambaro et al. conducted a complementation analysis with microcell-mediated chromosome transfer (MMCT) using a centric fragment of chromosome 3 (der3p12-q12.1) into the OV-90 EOC cell line lacking *VGLL3* expression [34]. Genetic characterization of the derived MMCT hybrids revealed that only the hybrid that contained an intact *VGLL3* locus exhibited alterations of tumorigenic potential in a nude mouse xenograft model and various in vitro growth characteristics. This indicates that persistent expression of *VGLL3* induced malignant tumors in nude mice and is fundamental to an aneuploidy-mediated phenotype. Dafou et al. found that chromosomes 4, 5, 6, 13, 14, 15, and 18 are frequently deleted in primary EOCs, and the authors used MMCT to establish the functional effects of transferring normal copies of these chromosomes into two epithelial EOC cell lines (TOV112D and TOV21G) [35]. The results revealed strong evidence of functional, neoplastic suppression for multiple hybrids in both cell lines on chromosomes 6 and 18, suggesting the presence of potential “tumor suppressor-like” genes within these chromosomes. However, comprehending aneuploidy presents a dual challenge. Firstly, the alteration of a specific chromosome disrupts the dosage of hundreds to thousands of genes, rendering it arduous to discern the pivotal genes that mediate the aneuploid phenotype. Secondly, there exists substantial heterogeneity in karyotypes (combinations of aneuploid chromosome copy numbers), giving rise to diverse effects on cells referred to as “karyotype-specific” effects. In this study, FISH technology with probes for CEP8 and c-Myc was employed to analyze EOC cell lines, tumor tissues, and peripheral blood samples from patients in order to investigate aneuploidy characteristics in ovarian cancer. Aneuploidy was detected across multiple chromosomes. The most significant alterations, however, were observed on chromosome 8, a prominent hallmark in ovarian cancer. Our research is specifically focused on investigating aneuploidy involving chromosome 8, as the identification of core genes associated with this phenomenon could significantly contribute to enhancing our understanding and treatment of cancer while also facilitating the discovery of novel therapeutic targets.

CTCs are cells shed from the primary tumor or metastases into the peripheral circulatory system [24]. Importantly, CTCs carry all or part of the genetic information of the primary tumor, which has made CTCs critical diagnostic markers and/or sources for tumor evaluation that aid in precision medicine. In our previous study, we used the SE-iFISH system with probes for CEP8 and CA125 or HE4 expression to identify CTCs [23]. This system is convenient for detecting the aneuploidy of Chr8, and the results indicated that among the 210 CTCs from 20 EOC patients, almost all CTCs showed Chr8 aneuploidy, which was consistent with single-cell sequencing and TCGA analysis.

FISH has been applied to study different attributes of the genome (i.e., pericentromeric regions, telomere analysis, chromosome painting, identification of euchromatic genomic loci, and simultaneous visualization of the complete set of chromosomes). FISH analysis with different probes for chromosomal centromeres has been widely used to detect tumor aneuploidy. In EOC, chromosome instability of Chr8 was a subject of focus for a long time. Wang et al. used interphase FISH in mechanically- and enzymatically-dispersed, frozen, invasive EOC samples to perform a Chr8 centromere study. The results showed that ovarian tumors exhibited polysomy 8; however, the presence of polysomy in Chr8 did not appear to be correlated with clinical presentation or disease progression [36]. Herein, we used a dual-color FISH in 32 FFPE EOC samples and found that almost all pathological types of EOC exhibited polysomy 8, while in serous EOC and OCCC (ovarian clear cell carcinoma), polysomy 8 was more significant. Using the median mean ploidy as the cut-off value, 16 patients were defined as having a high degree of Chr8 aneuploid, and the remaining patients showed a low degree of Chr8 aneuploid. We confirmed that polysomy 8 was not associated with any clinical characteristic covered, which was consistent with previous studies. This may be because of the limited sample numbers. 

The c-Myc gene is a multifunctional proto-oncogene located at 8q24, and c-Myc plays an important role in cell proliferation and the development of cancer. Amplification of c-Myc has been described in a variety of human cancers, including EOC. Multiple studies have shown an association between c-Myc amplification and poor outcomes in different cancers. In EOC, c-Myc amplification was shown to be associated with some clinical characteristics, while other studies showed no association [37]. However, the precise definition of c-Myc amplification remains elusive. Previous studies have encountered controversies regarding the characterization of c-Myc amplification due to challenges in distinguishing between mitotic events and numerical chromosomal instability on Chr8. Two distinct patterns of c-Myc amplification can be observed: either an amplification solely involving the c-Myc gene or a concurrent occurrence with Chr8 aneuploid change. Thus, the copy number of c-Myc may be influenced by aneuploidy of Chr8. The calibration of c-Myc amplification in accordance with the number of CEP8 probes is important. In our study, we performed FISH with probes for c-Myc and the centromeric region of Chr8 (CEP8), and c-Myc amplification was defined as ≥1.5 copies of c-Myc/CEP8. Amplification of c-Myc was associated with histology type, pathological grade, lymphatic metastasis, and positive expression of ER in EOC. The prognostic value of c-Myc amplification and polysomy 8 was not investigated because of the lack of clinical data. 

Single-cell methodologies provide a robust tool to complement and enhance the FISH-based pioneering investigations. Various molecular cytogenetic techniques with different levels of resolution and throughput are available for studying genome instability and copy number changes. Single-cell sequencing provides an effective means to investigate aneuploidy. In this study, we employed the single-cell multi-omics sequencing technology known as scCOOL-seq2, which enabled high-resolution detection of somatic copy number alterations (SCNAs), assessment of chromatin accessibility, whole-transcriptome profiling at the individual cell level, and direct analysis of subpopulations within larger heterogeneous populations [22]. This approach proves valuable for further exploring chromosome instability and identifying key genes.

Alterations in the copy number of specific chromosomes are expected to lead to upregulation or downregulation of genes located on the affected chromosomes. Simultaneous changes in gene expression levels across the entire chromosome can be employed to infer chromosome copy numbers. In our research, DNA contents or inferred DNA contents from transcriptional data in the centromere region of Chr8 were employed to distinguish different chromosome copy numbers in individual single cells. Using these criteria, enriched ovarian cancer (EOC) cells were classified into diploid tumor cells and polyploid tumor cells with various ploidy levels, including haploid, triploid, tetraploid, and multiploidy tumor cells on Chr8. Differential expression analysis was performed, revealing a total of 745 upregulated differentially expressed genes (DEGs). Gene ontology (GO) and Kyoto Encyclopedia of Genes and Genomes (KEGG) analyses identified the enrichment of these genes in cell division, cell cycle regulation, and terms related to the regulation of cell cycle progression. Analysis using The Cancer Genome Atlas (TCGA) dataset consisting of copy number variation (CNV), mRNA expression data, and clinical information also demonstrated that CNV genes predominantly amplified on Chr8 were enriched in the double-strand break repair pathway. These findings further validate our accuracy for distinguishing different ploidy states on Chr8 since they confirm that dysregulation of cell division and cell cycle processes strongly correlates with the occurrence of chromosomal segregation errors leading to chromosomal instability. Cell division is complex, with several proposed hypotheses explaining the generation of chromosomal instability, such as merotelic attachments, spindle assembly checkpoint defects, and chromosome cohesion defects being classical theories. Additionally, an intact DNA repair system is crucial for maintaining genetic stability since the accumulation of DNA damage contributes to increased chromosome instability. 

In both analyses of single-cell sequencing data and TCGA CNV data, the *PRKDC* gene emerged as the top-ranked hub gene associated with copy number variations (CNVs) and aneuploidy of Chromosome 8. *PRKDC* plays a pivotal role in mediating the initiation of non-homologous end-joining (NHEJ) repair, which is indispensable for both the causation and consequence of aneuploidy [38]. There is growing interest in unraveling the molecular mechanisms through which *PRKDC* facilitates tumor progression. Further analysis revealed an upregulation of *PRKDC* mRNA in epithelial ovarian cancer (EOC) compared to normal ovarian tissues from the GTEx database. This phenomenon was also confirmed through GEO data analysis. Although Kaplan–Meier plotter analysis indicated that *PRKDC* did not demonstrate prognostic value in the overall survival (OS) of EOC patients, it exhibited significance in terms of progression-free survival (PFS). PFS is more likely to reflect earlier stages of disease progression compared to OS. We included patients with lower pathological grades or earlier FIGO stages for survival analysis and observed that *PRKDC* overexpression was associated with poor prognosis in early-stage EOC patients or those with low-grade tumors. The analysis of TCGA database also revealed that patients exhibiting amplification in *PRKDC* copy number experience a more unfavorable prognosis. Bioinformatics analysis of *PRKDC* indicated that *PRKDC* plays an important role in epithelial ovarian cancer progression.

It is worth noting that the upregulation of *PRKDC* expression may arise as a consequence of amplified copy number variation on chromosome 8 (Chr8); however, it also has the potential to contribute to these copy number alterations. Further investigation is warranted to ascertain whether *PRKDC* CNV is a consequence or driver of chromosomal instability. To investigate whether the alteration in *PRKDC* is caused by Chr8 CNV, we analyzed TCGA database and observed a strong correlation between the copy number variation (CNV) status of *PRKDC* and its mRNA level. Functional analysis further confirmed the upregulation of *PRKDC* in epithelial ovarian cancer. Enriched single cells with higher expression levels of *PRKDC* exhibited more CNVs on Chr8, while ovarian cancer cells transfected with siRNA or dsRNA targeting *PRKDC* mRNA also showed affected CNVs on Chr8, providing additional evidence for the influence of *PRKDC* expression on CNV occurrence at Chr8. However, this may be attributed to the upregulation of *PRKDC* expression, which in turn facilitates enhanced global genetic instability. To investigate the broader spectrum of this instability, we conducted a comprehensive analysis of overall chromosomal alterations in two distinct cell populations exhibiting differential levels of *PRKDC* expression. The results indicate that there was no statistically significant difference in total DNA CNV values between the two groups, which indicated that *PRKDC* predominantly influenced chromosome instability on chromosome 8 rather than exerting a global effect on the entire genome. Previous studies have demonstrated that increased global genome instability results in elevated DNA damage. To further validate the impact of *PRKDC* expression on chromosome 8 copy number alterations rather than global genome instability, γ–H2AX immunofluorescence was employed to detect DNA damage in cells. The findings confirmed that *PRKDC* did not significantly affect whole-genome copy number changes and that its effect on chromosome 8 copy number was independent of its influence on the entire genome. Meanwhile, we investigated whether CNV at Chr8 could affect the expression level of *PRKDC* by dividing 10 patients enrolled in our single-cell sequencing into two groups based on their degree of CNV at chromosome 8. Our results demonstrated that the expression level of *PRKDC* can indeed be affected by CNV at Chr8. Survival analysis further indicated that CNV involving *PRKDC* serves as a marker for poor progression-free survival (PFS) and overall survival (OS).

*PRKDC*, also named DNA-PKcs (protein kinase DNA-activated catalytic subunit), is a key component of the nonhomologous end-joining (NHEJ) pathway for DNA damage response and double-strand break (DSB) repair. *PRKDC* is a member of the phosphatidyl inositol 3 kinase-like (PIKK) protein kinase group. It functions in NHEJ repair as a catalytic subunit that binds with Ku70 and Ku80 to form the DNA-PK complex and then recruits the XRCC4 /ligase 4 complex to directly rejoin the two ends of a DSB. DNA DSBs are the most serious DNA damage in cells, and repair of DSBs generally occurs through two mechanisms: homologous recombination (HR) repair and non-homologous end-joining (NHEJ) repair. HR is an accurate repair mechanism that requires homologous sequence templates and can only occur in cells in the G2/S phase. NHEJ does not depend on the existence of the DNA template and responds rapidly throughout the cell cycle, thereby representing a mistake-prone repair mechanism that can cause chromosome instability. An aberrant functional DNA repair system was proven to be strongly associated with the generation of aneuploidy and may drive the progression of cancer via specific mechanisms, including by mediating the aneuploid phenotype [39]. Aberrant expression or mutations of the *PRKDC* gene have been observed in various human cancers. Elucidating the molecular mechanisms underlying *PRKDC* promotes tumor progression is a primary focus of ongoing research efforts. Additionally, *PRKDC* has been implicated in tumor cell resistance and plays a crucial role in regulating cell cycle progression and chromosomal segregation. In ovarian cancer, further functional experiments are required to determine whether increased *PRKDC* influences the NHEJ pathway, while elucidation of the underlying molecular mechanism linking *PRKDC* to chromosome instability in ovarian cancer is necessary for its potential application. Further investigation is warranted to explore the association between the *PRKDC*-mediated DNA repair system and aneuploidy generation [40], which would enhance our understanding of this complex biological process.

Collectively, our findings suggest that copy number amplification contributes to the upregulation of *PRKDC* expression in EOC. Both *PRKDC* and its mediated DNA double-strand break repair pathway play crucial roles in driving the evolution of the Chr8 aneuploidy phenotype and promoting disease development. Further investigation into the intricate functions of *PRKDC* associated with Chr8 aneuploidy will help elucidate their detailed mechanisms underlying EOC pathogenesis, providing novel insights for prognostic biomarker discovery and identification of molecular targets for EOC therapy.

## 4. Materials and Methods

### 4.1. SCNA Estimation from Published Single-Cell Multi-Omics Sequencing Data

We downloaded our published single-cell multi-omics sequencing raw data of 14 ovarian cancer patients and 3 healthy controls for single-cell somatic copy number alterations (SCNA) analysis (GSA: HRA000360). The preprocessing was performed as previously described [22]. Briefly, using Trim Galore (v0.3.3) (https://github.com/FelixKrueger/TrimGalore, accessed on 8 October 2021) to remove random primer sequences, adaptor sequences, and low-quality bases. Then, Bismark (v0.7.6) (https://github.com/FelixKrueger/Bismark, accessed on 8 October 2021) was used to map clean reads to the *human* reference genome (hg19, UCSC) with a paired-end and nondirectional module. Finally, the cell without duplicates was used for the SCNA estimate.

We used Ginkgo to estimate SCNAs from single-cell genome sequencing data, including binning uniquely mapping reads into variable-length intervals across the genome (with a median length of 1 Mb), excluding centromere and telomere regions, normalizing for GC bias, chromosome segmentation, and finally assigning integer copy numbers to each segment.

### 4.2. The Estimation of Aneuploidy on Chromosome 8 from Single-Cell Genome Sequencing Data

A centromere probe for Chr8p11.1-8q11.1 of FISH has been designed to determine the aneuploidy on chromosome 8. To correspond to the loci that the centromere probe used, we only examined the copy number variation in chr8:42754568-47889815, which we used to represent the ploidy of Chr8 in a single cell. After genome-wide SCNAs analysis from Ginkgo, the copy number of Chr8:42754568-47889815 in the single cell was used for subsequent analysis.

### 4.3. TCGA Data Processing Identification of CNV

A total of 589 ovarian cancer patients in TCGA database with CNV and clinical data were included in this study for the analysis of somatic copy number alterations in ovarian carcinoma samples. Following screening, a total of 578 samples exhibiting both *PRKDC* copy number variations and clinical data were utilized for subsequent clinical analysis. The Genomic Identification of Significant Targets in Cancer algorithm (GISTIC) was used to identify the loss and gain levels of copy number of specific genes. GISTIC is an algorithm used to identify regions of variation that are more likely to trigger cancer pathogenesis. Taking the number of copies greater than 1 as the threshold of copy amplification and less than −1 as the threshold of copy loss, we calculated the ratio of copy amplification and loss for each gene and observed their distribution in the genome. A value of 0 equates to no change in copy number; a positive 1 or 2 means an amplification, and a negative 1 or 2 means a deletion; 1 or 2 signifies the level of the amplification of the CNV, with 2 signifying a higher level of amplification. For this study, we combined the values 1 or 2 to categorize the CNV as amplification, −1 or −2 as deletion, and 0 as no change. All the data are publicly available and open-access. We used only anonymous statistical gene expression. To evaluate the clinical significance of the CNV status, the EOC cohort was divided into two subgroups: “with CNV the *PRKDC* genes” and “without CNV”. 

### 4.4. TCGA and GEO Data Processing Identification of mRNA Expression of PRKDC

A total of 374 EOC samples from TCGA database and 180 normal ovarian tissues from the GTEx database as control were included in this study. Gene expression data from EOC tissues and normal fallopian tube epithelial (FTE) or ovarian surface epithelium (OSE) tissue samples were downloaded from GEO. Validation of *PRKDC* expression was performed using three GEO databases, namely GSE69428 (n = 20), GSE40595 (n = 38), and GSE18520 (n = 63). The mRNA level of *PRKDC* was analyzed with the “limma” package. 

### 4.5. Detection of CTC 

The detailed method and screening cohort were shown in our previous research [23,24]. Briefly, the SE-iFISH system was used to identify circulating tumor cells. A total of 20 patients diagnosed with ovarian cancer who were admitted to Peking University People’s Hospital between May 2018 and December 2020 were included in this study. At first, SE was performed according to the manufacturer’s instructions in order to eliminate the white blood cells and red blood cells in PB (Peripheral Blood). Samples were subsequently immunostained with fluorescence-labeled monoclonal antibodies of lymphocytes (anti-CD45-Alexa594), endothelial cells (anti-CD31-Cy5), and TMs of EOC (anti-CA125-Cy7, anti-HE4-Alexa488). Next, samples were coated on the slides and subjected to a Vysis CEP 8 Spectrum Orange Direct Labeled Fluorescent DNA Probe Kit (Abbott Molecular, Des Plaines, IL, USA). Finally, automated Metafer-i·FISH^®^ CTC 3D scanning and image analyzing systems were used to scan coated slides containing CTCs. 

### 4.6. Cell Culture

*Human* ovarian cancer cell lines SKOV3, A2780, ES2, SKOV3-IP, and CAOV-3 were obtained as described previously. These ovarian cancer cell lines were cultured in McCoy’s 5A or RPMI 1640 (Gibco, New York, NY, USA). hEM15A is an immortalized stromal cell line derived from eutopic endometrial tissue of EM patients, which was established by our lab and preserved in the China Center for Type Culture Collection and Cell Resource Center of Peking Union Medical. hEM15A cells were cultured in Dulbecco s modified Eagle s medium (DMEM)/F12 (Gibco, New York, NY, USA) supplemented with 15% fetal bovine serum (FBS, Gibco) and 1% penicillin/streptomycin. All cells were maintained at 37 °C in a humidified atmosphere with 5% CO_2_ atmosphere.

### 4.7. Tumor Specimens and Fluorescence In Situ Hybridization

A total of 44 tissue samples from 32 patients diagnosed with epithelial ovarian cancer who underwent surgical treatment at Peking University People’s Hospital between December 2005 and December 2019 were included in this study. Previously untreated, the primary tumor was excised during cytoreductive surgery, fixed in formalin, and then embedded in paraffin. Unstained tissue sections, 5 µm in thickness, were prepared on charged glass slides. The number of copies of c-Myc and chromosome 8 in unstained slides was quantified through Dual-label FISH. Briefly, unstained sections were baked overnight at 60 °C, deparaffinized, dehydrated, treated for 10 min with 4% (*w*/*v*) pepsin at 45 °C, denatured for 10 min at 90 °C, hybridized for 12 to 16 h at 37 °C with a probe cocktail consisting of a centromere probe to Chr8p11.1-q11.1 (CEP8) directly labeled with Spectrum Aqua and a c-Myc region probe directly labeled with Spectrum Orange (Vysis Inc., Naperville, IL, USA). The slides were post-washed for 5 min with 2×SSC buffer (0.3 M NaCl and 30 mM sodium citrate tribasic dihyrate, pH 7.0) at 72 °C and counterstained with 4′,6′-diamidino-2-phenyindole (DAPI). Green staining for chromosome 8 and red staining for c-Myc was visualized using a Zeiss Axiophot fluorescence microscope (Carl Zeiss MicroImaging, Inc., Thornwood, NY, USA) with appropriate filters and an Applied Imaging Cytovision system (Pittsburgh, PA, USA), and quantified in at least 100 tumor cells per case by one or two technicians (CC and GV; see Acknowledgements) working under the direct supervision of one of the authors (JKB). 

### 4.8. Analysis of Gene Expression Profiles by Metascape

The enrichment of Gene Ontology (GO) and Kyoto Encyclopedia of Genes and Genomes (KEGG) were analyzed by Metascape^®^ (http://metascape.org, accessed on 15 October 2023) to find the aneuploid-related genes in the different genes list screened by the single-cell sequencing. Metascape is a gene annotation and analysis resource that updates the information monthly, and the last update was on 11 November 2018. GO analysis is a major bioinformatics tool commonly used to interpret high-throughput genomic or transcriptome data to unify the representation of gene and gene product properties across all species. KEGG is a database resource for molecular-level information, especially genome sequencing and other high-throughput large-scale molecular data for understanding the advanced functions and utilities of biological systems, such as cells, organisms, and ecosystems, and experimental techniques. 

### 4.9. PPI and Hub Gene Screening

Online websites STRING and cytoscape were used to build the PPI network and analyze the functional pathways. Cytoscape functioned as a network graph, with differentially expressed molecules represented as nodes and intermolecular interactions represented as links, that is, edges, between nodes. In this study, the combined score represented intermolecular interactions, and the protein pairs with a combined score > 0.4 were selected to construct the PPI network. Nodes were drawn in different sizes and colors, which represented the node degree and the regulation (up or down), respectively. The cytohubba is a plugin of cytoscape. It was widely used and essential for us to explore the most important node in diverse biological networks. In this study, we used Degree, Density of Maximum Neighborhood Component (DNMC), and Maximal Clique Centrality (MCC) to identify the candidate hub genes.

### 4.10. Survival Analysis 

The association between the mRNA expression levels of the *PRKDC* gene and OS and PFS was analyzed using an online KM-Plotter database using the gene expression data and the survival information of patients with ovarian cancer downloaded from the GEO (https://www.ncbi.nlm.nih.gov/pubmed/20020197, accessed on 15 October 2023). A total of 1656 patients diagnosed with ovarian cancer were included in the overall survival analysis, among whom 380 patients had pathological grades 1 and 2. Additionally, there were 135 patients classified as FIGO stage I and II. A total of 1435 patients diagnosed with ovarian cancer were included in the progression-free survival analysis. Cohorts of patients were split by median expression values through auto-select best cut-off. 

### 4.11. Immunohistochemistry Assay

Ten tissue samples of epithelial ovarian cancer patients who underwent single-cell sequencing were selected for immunohistochemistry (OC04, OC05, OC08, OC09, OC14, OC06, OC07, OC11, OC13, OC16). Fresh tissues from tumor xenograft were fixed with 4% paraformaldehyde for 24 h before dehydrated and embedded in paraffin. Tissue sections were torrefied for 1 h and dewaxed with xylene and ethyl alcohol. The microwave antigen retrieval technique was used to repair the antigen. Sections were incubated with 3% H_2_O_2_ for 20 min to block endogenous peroxidase activity and incubated with goat serum for 30 min to block non-specific antigens. Then, sections were incubated with primary antibodies (*PRKDC*, 1:400) overnight at 4 °C. After incubation with biotin-labeled goat anti-rabbit IgG polymer and horseradish enzyme-labeled streptomycin for 30 min, respectively, positive signals were detected with DAB reagent and quantified by Image-Pro Plus software 6.0 (Media Cybernetics, Rockville, MD, USA). The same setting was used for all the analyzed tissues to be accurate for the staining reading. Integrated optical density (IOD) and size of the total area were measured in each field, and the staining score was formulated as IOD/size. 

### 4.12. Western Blot Analysis

Tissue proteins were obtained from 10 ovarian cancer patients who underwent single-cell sequencing (OC04, OC05, OC08, OC09, OC14, OC06, OC07, OC11, OC13, OC16). As for cellular proteins, a total of 2 to 5 × 10^6^ cells were lysed on ice for 1 min with 1mL lysis buffer plus protease inhibitor (Sigma Aldrich, St. Louis, MO, USA). After centrifugation at 1 × 10^4^ rpm for 15 min, the lysate was boiled with 5X sample buffer, then separated by sodium dodecyl sulfate-polyacrylamide gel electrophoresis and transferred to a polyvinylidene difluoride membrane. Immunoblotting was performed by chemiluminescence and visualized. Antibodies used for immunoblotting were as follows: PRKDC (CST, Cat. 38168); GAPDH (CST, Cat. 5174).

### 4.13. Quantitative Real-Time Transcription-Polymerase Chain Reaction

Total RNA of cells was extracted, and concentration and purity were detected using a spectrophotometer (Thermo Fisher Scientific Inc., Waltham, MA, USA). Then, the RNA (3000 ng/20 μL reaction system) was transcribed into cDNA. The PCR reaction was performed on a StepOne™ PCR amplifier (Bio-Rad Laboratories, Hercules, CA, USA) with SYBR-green (TaKaRa Biotechnology, Shiga, Japan) in a 10 μL reaction system, qPCR reaction was performed on the Bio-Rad C1000 Real-time fluorescence thermal cycler (Bio-Rad Laboratories, USA), using the following cycling conditions: Initiation at 95 °C for 10 min; amplification for 35 cycles, with denaturation at 95 °C for 30 s; annealing at 56 °C for 30 s; and elongation at 72 °C for 30 s. A final extension at 72 °C was performed for 10 min, and GAPDH mRNA level was used for normalization.

### 4.14. Small Interfering RNA Transfection

Three different siRNA targeting PRKDC mRNA (siPRKDC-1#, siPRKDC-2#, and siPRKDC-3#) and non-targeting control(siCTRL) were synthesized by GenePharma Company. Ovarian cancer cells were seeded on 6-well plates 24 h before siRNA transfection. Lipofectamine 3000 (Invitrogen, Inc., Carlsbad, CA, USA) was incubated with opti-mem (Gibco-BRL, Grand Island, NY, USA) ahead of the mixture between siRNA for *PRKDC* (150 pmol per well) or siCTRL (150 pmol per well) and opti-mem. The above solutions were mixed and incubated at room temperature for 15 min. Then, they were dropwise added to 1640 supplemented with 10% FBS. After 24 h, the medium was replaced with fresh 10% FBS/1640.

### 4.15. Small Activating RNA (saRNA) Design and Transfection

A 21-nucleotide (nt) dsRNA targeting the *PRKDC* promoter at position-9874 relative to its transcription start site (dsPRKDC-2-9874) was used to activate *PRKDC* expression. The sequences of saRNA-PRKDC-1(dsPRKDC-2-9874) used in this experiment were as follows: Sense, 5′-GCTACTTGGTGTTGGACTTGG-3′ and antisense, 5’-CCAAGTCCAACACCAAGTAGC-3′. Transfection of dsRNA was carried out using Lipofectamine RNAiMax (Invitrogen) or according to the reverse transfection protocol of the manufacturer’s instructions. After a 5- to 6-h incubation, transfection complexes were removed and replaced with a fresh growth medium containing either chemical compounds or dimethyl sulfoxide (DMSO).

### 4.16. γ–H2AX Foci Staining

γ–H2AX foci staining cells were seeded on coverslips at a density of 5 × 10^4^. After each time point, cells were fixed with 4% paraformaldehyde and permeabilized with 0.25% TritonX for 5 min. Samples were blocked using 4% Bovine Serum albumin, then incubated with anti-phospho-Histone H2A.X antibody (1:1000) (Sigma Aldrich, USA; #05-636) for 1 h. After washing, samples were incubated with Alexa-Fluor 647 goat anti-mouse antibody for 30 min. Nuclei were counterstained with 1 µg/mL of 4′,6′-diamidino-2-phenyindole (DAPI), and the coverslips were mounted and then imaged using a confocal microscope. ImageJ software was used to quantify DNA damage.

### 4.17. Statistical Analysis

SPSS software (Version 24.0; IBM Corp, New York, NY, USA) and GraphPad Prism 8 (GraphPad Software, La Jolla, CA, USA) were used for all statistical analyses. One-way analysis of variance (ANOVA) and unpaired Student’s *t*-test were used to analyze the differences between two groups and among several groups of separate data. The correlation was examined using Spearman correlation analysis. The correlation of CEP8 positivity with clinical pathologic characteristics was examined using Fisher’s exact test. The classification data were expressed as percentiles. Pearson’s correlation analysis was used to determine the correlation; *p*-values < 0.05 were considered statistically significant.

## 5. Conclusions

In summary, the amplification of CNV and the presence of aneuploidy in chromosome 8 were identified as significant characteristics of ovarian cancer. *PRKDC* and the mediated non-homologous end-joining (NHEJ) pathway may play a pivotal role in the induction of aneuploidy on chromosome 8, driving the progression of ovarian cancer.

## Figures and Tables

**Table 1:** Relevant patient characteristics for fluorescence in situ hybridization (FISH) were examined.

Characteristics	Cohort (n = 32) No. of Patients (%)
Age	
<50	12 (37.5%)
≥50	20 (62.5%)
Histology Type	
HGSOC	16 (50%)
LGSOC	5 (15.625%)
OCCC	4 (12.5%)
ENOC	5 (15.625%)
MOC	2 (6.25%)
FIGO stage	
Ⅰ	8 (25%)
Ⅱ	3 (9.375%)
Ⅲ	16 (50%)
Ⅳ	5 (15.625%)
Pathological grade	
G1	16 (50%)
G2	11 (34.375%)
G3	5 (15.625%)

Abbreviation: HGSOC, High-grade serous ovarian cancer; LGSOC, Low-grade serous ovarian cancer; OCCC, ovarian clear cell carcinoma; ENOC, Endometrioid ovarian cancer; MOC, mucinous ovarian carcinoma; FIGO, International Federation of Gynecology and Obstetrics.

**Table 2:** The association between clinicopathologic parameters and CEP8 status in a full cohort of ovarian cancer.

Characteristics	CEP8		*p*-Value
	Low (n = 16)	High (n = 16)	
Age	49.56 ± 3.202	53.31 ± 1.852	0.319
Histology Type			0.106
HGSOC	8 (50.0)	8 (50.0)	
LGSOC	2 (12.5)	3 (18.7)	
OCCC	0	4 (25.0)	
ENOC	4 (25.0)	1 (6.3)	
MOC	2 (12.5)	0	
FIGO stage			0.500
I+II	6 (37.5)	5 (31.3)	
III+IV	10 (62.5)	11 (68.7)	
Pathological grade			0.638
G1 + G2	8 (50.0)	8 (50.0)	
G3	8 (50.0)	8 (50.0)	
Lymphatic metastasis			0.723
Yes	9 (56.3)	8 (50.0)	
No	7 (43.2)	8 (50.0)	
Ascites			0.476
Yes	6 (37.5)	8 (53.8)	
No	10 (62.5)	8 (46.2)	
ER			0.394
Positive	14 (87.5)	11 (73.3)	
Negative	2 (12.5)	4 (23.7)	
P53			0.273
Positive	8 (53.3)	12 (75.0)	
Negative	7 (46.7)	4 (25.0)	

Abbreviation: HGSOC, High-grade serous ovarian cancer; LGSOC, Low-grade serous ovarian cancer; OCCC, ovarian clear cell carcinoma; ENOC, Endometrioid ovarian cancer; MOC, mucinous ovarian carcinoma; ER, estrogen receptor; FIGO, International Federation of Gynecology and Obstetrics.

**Table 3:** The association between clinicopathologic parameters and c-Myc/CEP8 status in a full cohort of ovarian cancer.

Characteristics	c-Myc/CEP8		*p*-Value
	<1.5 (n = 19)	≥1.5 (n = 13)	
Age	49.68 ± 2.912	54.50 ± 1.540	0.225
Histology type			0.016
HGSOC	5 (26.3)	11 (84.6)	
LGSOC	5 (26.3)	0	
OCCC	3 (15.8)	1 (7.7)	
ENOC	4 (21.2)	1 (7.7)	
MOC	2 (10.5)	0	
FIGO stage			0.450
I+II	8 (42.1)	3 (23.1)	
III+IV	11 (57.9)	10 (76.9)	
Pathological stage			0.003
G1 + G2	14 (73.7)	2 (15.4)	
G3	5 (26.3)	11 (84.6)	
lymphatic metastasis			
Yes	6 (31.6)	9 (69.2)	0.041
No	13 (68.4)	4 (30.8)	
Ascites			
Yes	11 (57.9)	7 (53.8)	0.553
No	8 (42.1)	6 (46.2)	
ER			
Positive	12 (66.7)	13 (100)	0.025
Negative	6 (33.3)	0	
P53			
Positive	12 (66.7)	8 (61.5)	0.532
Negative	6 (33.3)	5 (38.5)	

Abbreviation: HGSOC, High-grade serous ovarian cancer; LGSOC, Low-grade serous ovarian cancer; OCCC, ovarian clear cell carcinoma; ENOC, Endometrioid ovarian cancer; MOC, mucinous ovarian carcinoma; ER, estrogen receptor; FIGO, International Federation of Gynecology and Obstetrics. Note: Ratio of c-Myc/centromere 8/cell ≥ 1.5.

**Table 4:** The association between clinicopathologic parameters and PRKDC CNV status in a full cohort of ovarian cancer.

Characteristics	PRKDC		*p*-Value
	Without (n = 401)	With (n = 177)	
Age			0.026
<50	222 (55.4)	81 (45.8)	
≥50	179 (44.6)	96 (54.2)	
Pathological stage			<0.001
G1 + G2	38 (9.5)	9 (5.2)	
G3	352 (87.8)	89 (51.4)	
G4	11 (2.7)	75 (43.4)	
Unknown	0	4	
FIGO stage			<0.001
I+II	61 (15.2)	14 (8.0)	
III	340 (84.8)	147 (84.5)	
IV	0	13 (7.5)	
Unknown	0	3	
Venous invasion			<0.001
NO	59 (39.3)	14 (100)	
Yes	91 (60.7)	0	
Unknown	251	163	
Lymphatic invasion			<0.001
NO	63 (34.1)	20 (55.6)	
Yes	122 (65.9)	16 (44.4)	
Unknown	216	141	

Note: With represents patients that had copy number variation of PRKDC. Without represents patients that did not have copy number variation of PRKDC.

## Data Availability

The analyzed data sets generated during the study are available from the corresponding author upon reasonable request. The data are not publicly available due to [legal or privacy concerns, necessitating the maintenance of confidentiality for the patients involved in the study].

## Supplementary Materials

The following supporting information can be downloaded at: https://www.mdpi.com/article/10.3390/ijms25094825/s1.
