# Supplementary material for: PRKDC-Mediated NHEJ May Play a Crucial Role in Aneuploidy of Chromosome 8-Driven Progression of Ovarian Cancer
Source: Int J Mol Sci. 2024 Apr 28;25(9):4825. doi: 10.3390/ijms25094825 (PMC11084440; doi:10.3390/ijms25094825)
Supplement: Supplementary file 1 [file ijms-25-04825-s001.zip › ijms-2888444-supplementary.pdf]

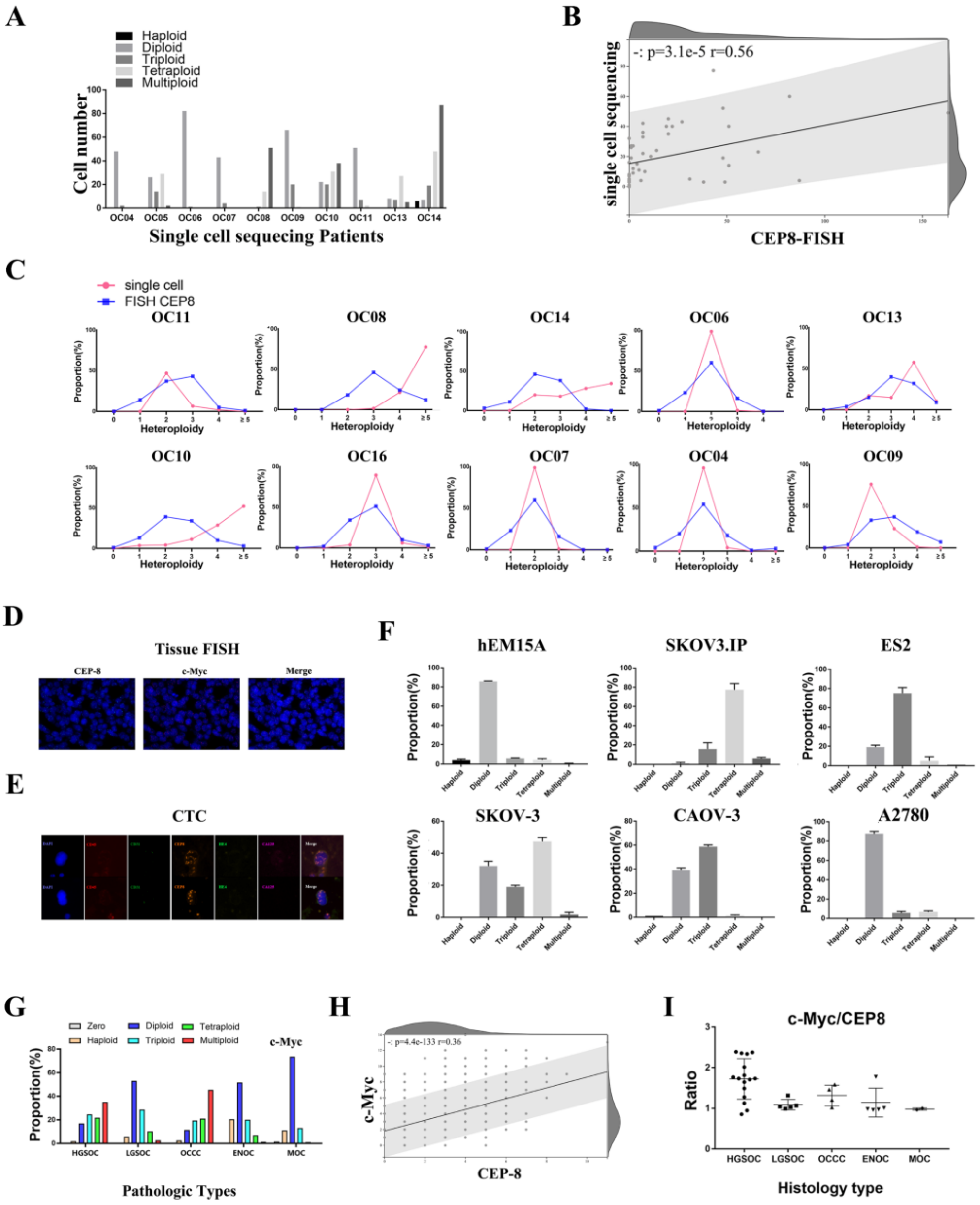

Figure S1. The relevance between CEP-8 FISH and single cell sequencing and the relevant results of c-Myc. (*A*) The number of sub-group of different chr8 aneuploid cells of patients of single-cell sequencing. (*B*) The correlation analysis between CEP-8 FISH and chr8 sub-classification of single-cell sequencing. (*C*) The proportion of different chr8 ploidy cells of CEP-8 FISH and single-cell sequencing. (*D*) The typical pictures of CEP-8 and c-Myc FISH of ovarian cancer tissue. (*E*) The typical pictures of CTCs captured by SE-iFISH. (*F*) The proportion of different ploidy of chromosomes 8 detected by CEP8 FISH in different ovarian cancer cell lines. (*G*) The proportion of different ploidy of c-Myc detected by c-Myc FISH in different pathological types of ovarian cancer. (*H*) Spearman correlation analysis of CEP8-FISH and c-Myc FISH. The horizontal axis represents the chromosome 8 status, and the vertical axis represents c-Myc status. On the upper side and the right side are the Gray bar chart showing the distribution trend of probe of CEP8 and c-Myc. (*I*) The ratio of c-Myc/CEP8 in different pathological types of ovarian cancer.

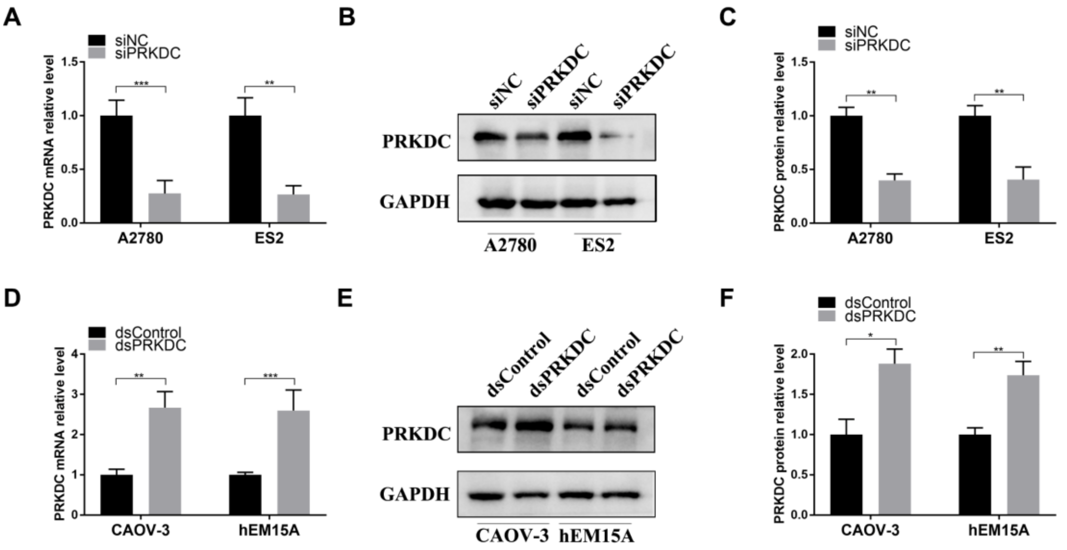

Figure S2. The mRNA and protein levels of PRKDC in cells were detected after transfected with siRNA and saRNA. (A) Expression of PRKDC mRNA levels were assessed by real-time PCR in A2780 and ES2 after transfected with siRNA. (B) Expression of PRKDC protein levels were assessed by Western Blot in A2780 and ES2 after transfected with siRNA. (C) Quantification of PRKDC protein relative level. (D) Expression of PRKDC mRNA levels were assessed by real-time PCR in CAOV-3 and hEM15A after transfected with saRNA. (E) Expression of PRKDC protein levels were assessed by Western Blot in CAOV-3 and hEM15A after transfected with saRNA. (F) Quantification of PRKDC protein relative level
